# Experiences of Students with Disabilities Enrolling in Higher Education: A Qualitative Study in the United Arab Emirates

**DOI:** 10.12688/f1000research.158481.1

**Published:** 2025-01-02

**Authors:** Muhammad Arsyad Subu, Richard Mottershead, Ahmed Mahmoud Sallam, Jacqueline Maria Dias, Janisha Kavumpurath, Fatma Refaat Ahmed, Heba Hesham Hijazi, Mini Sara Abraham, Alounoud Mohamed Al Marzouqi, Vidya Seshan, Shahd Mohammed Elabed, Ahmad Rajeh Saifan, Syed Azizur Rahman, Nabeel Al Yateem

**Affiliations:** 1 Faculty of Nursing and Midwifery, Universitas Binawan, Jakarta, Indonesia; 2 Nursing Department, College of Health Sciences, University of Sharjah, Sharjah, Sharjah, United Arab Emirates; 3 Disability Resource Center, University of Sharjah, Sharjah, Sharjah, United Arab Emirates; 4 Department of Health Care Management, College of Health Sciences, University of Sharjah, Sharjah, Sharjah, United Arab Emirates; 5 Department of Health Management and Policy, Faculty of Medicine, Jordan University of Science and Technology, Irbid, Jordan; 6 College of Nursing, Yarmouk University, Irbid, Jordan

**Keywords:** students with disabilities, university, higher education, qualitative, United Arab Emirates.

## Abstract

**Background:**

Higher education is experiencing an increase in students with disabilities, necessitating considering their interests and well-being when preparing educational services. Students with disabilities make up one of the most significant minority groups among students in terms of diversity.

**Aims:**

This study explored the experiences of students with disabilities at a university in the United Arab Emirates. It also aimed to understand students with disabilities’ social and academic experiences and the factors affecting their university persistence.

**Methods:**

We used a descriptive qualitative research approach to understand how students with disabilities experience the phenomenon under study. Twenty-five students participated, chosen via purposeful sampling. The data was collected using semi-structured interviews, which were analyzed using content analysis.

**Results:**

We found four themes that emerged from this study: (1) perceptions about the university, (2) socialization and integration, (3) barriers to the university journey, and (4) support for the journey.

**Conclusion:**

We found that students with disabilities indicated positive and negative perspectives and experiences. We recommend modifying the physical, social, and educational environments to support the education of students with disabilities. Future studies should employ a more varied sample and include academics and students to collect different perspectives while stressing the students’ experiences.

## Introduction

Disability is the condition in which a person’s ability to engage in activities is limited due to their interactions with the environment. Students with disabilities continue to face systematic disadvantages in comparison to their non-disabled peers. Hence, educational practitioners and policymakers must fully understand the intricacy of the encounters of impaired students ( Brewer et al., 2023). Some types of disabilities include hearing loss, movement problems, mental health issues, communication problems, and learning difficulties. The enrollment of students with disabilities in higher education has increased ( National Center for Education Statistics, 2022).

In order to combat the systemic discrimination that people with disabilities experience, structural interventions are necessary for students with disabilities ( Ostiguy-Finneran et al., 2018). The rights of students with disabilities in higher education are becoming more widely known internationally ( Taneja-Johansson, 2021), particularly considering an increase in the proportion of university students with disabilities ( Zhang et al., 2018). Higher education institutions must empower the disabled population, emphasizing student accessibility as these students grow. For students with disabilities, getting into higher education remains challenging ( Fernández-Batanero et al., 2022). Students with disabilities have a range of difficulties, such as physical, sensory, learning, attention, and communication challenges ( Burgstahler, 2020).

The educational environment and instructors’ capacity to modify it to be congruent with students’ skills are problems for students with disabilities. They continue to get unfair treatment while attending universities ( García-González et al., 2020). Due to difficulties with accommodation offered by universities, students with disabilities continue to face barriers when trying to access possibilities for a fair education ( Ehlinger & Ropers, 2020; García-González et al., 2020; Magnus & Tøssebro, 2014). They encounter problems, including prejudice and stigmatization, occasionally hindering their learning capacity ( Carroll et al., 2020; Subu et al., 2024). Physical environment and academic and administrative procedures are critical barriers to inclusive educational practices that university students with disabilities must overcome ( Algolayalt et al., 2023). Many students with intellectual disabilities struggle to finish their coursework and lack the intellectual and social skills necessary to fit in the universities ( Prohn et al., 2019) despite the multiple university education programs available to them.

The United Arab Emirates (UAE) adopted the international movement regarding the rights of individuals with disability by introducing a federal law in 2006 to govern the rights of people with disabilities, especially those related to education. Federal Law No. 29 of 2006 is the first legislation in the UAE to safeguard the rights of individuals with disabilities. According to Article 12 of the law, the country ensures that individuals with special needs have equal access to education in all educational institutions, including regular and special classes. This includes providing a curriculum in sign language, braille, or other suitable methods ( United Arab Emirates Government, 2023). Because of this, the UAE Ministry of Education launched a new program to include students with special needs in regular classrooms ( Gaad & Almotairi, 2013).

Special education is specifically designated for children who have a disability, problem, or any other circumstance that affects their capacity to learn or perform academically. In addition, people with disabilities are also ensured equitable chances and appropriate adjustments to pursue their studies in mainstream and specialized classrooms ( UNESCO, 2018). The UAE government has built assistance centers around the UAE to monitor the academic success of people with special needs before and after school integration. These centers offer various services, including assessing students with disabilities and learning challenges, making recommendations, helping parents navigate their child’s situation, and advising on appropriate support services for their children ( UAE government, 2023).

Within the Disability-Diversity (Dis)Connect model ( Aquino, 2016), two forms of disability diversity manifest in university settings. These models show how students with disabilities can be included and deal with comparable problems as students who identify with other diverse memberships or who cannot. Every experience a student has with disability and diversity is related to how they view their impairment’s role in their life and the university setting. First, *Disability-Diversity Disconnect:* Because they find it difficult to integrate their disability into the higher education environment and because their experiences differ from those of students who identify with other diversity memberships, students with disabilities do not want to participate fully (or intentionally) in the postsecondary environment and do not integrate. Students of this kind have a difficult time adjusting to life in a university setting. The student does not believe that disabilities are accepted in college, especially when they are a part of student diversity. Second, *Disability-Diversity Connect:* The student with a disability views the disability as just one of many elements that make up their identity and place in a postsecondary environment. Furthermore, one may consider disability in the postsecondary setting a part of diversity. According to the student, disability is fully integrated into the diversity of students and is a crucial aspect of the postsecondary environment. Positive experiences with self-development, a desire to engage with the postsecondary community, and the accomplishment of social and academic objectives all contribute to feelings of disability and diversity inclusion.

## Research aims

An extensive literature review indicates limited information regarding the experiences of students with disability currently enrolled in higher education institutions in the UAE.

This study explored the experiences of students with disabilities presently enrolled in higher education institutions in the UAE. Understanding the disparity in enrollment and degree completion rates for students with disabilities is essential for researchers, policymakers, and faculty members ( Erickson et al., 2014). The findings of this study may also assist educators in creating successful techniques to address the obstacles faced by students with disabilities. Organizations and decision-makers may use the results of this study to develop plans for meeting the social and academic requirements of university students with disabilities.

## Research questions

The main research question is: What are the experiences of disabled students at a higher education or university?

### Specific research questions

1. What do students with disabilities identify as positive and negative experiences studying in the university setting?
2. How do the negative and positive experiences make students with disability feel?
3. What do students with disabilities see as barriers and facilitators to their access to the university?

## Methods

### Design

The purpose of this study was to explore the experiences of students with disabilities enrolling at the University of Sharjah in the United Arab Emirates. This study adopted qualitative content analysis methods. Using qualitative methods allowed for an in-depth exploration of students’ lived experiences, providing a deeper understanding of their perspectives, challenges, and unmet needs. Researchers can better comprehend the complex and comprehensive nature of the topic they are studying by using the qualitative research design ( Creswell, 2012). According to Creswell and Poth (2018), qualitative research focuses on the substance and meaning of the world using interpretive and naturalistic methodologies. Content analysis is an interpretive process that focuses on the topic and background and examines similarities and differences between and within various text areas ( Graneheim & Lundman, 2004).

### Setting and participants

This research was conducted at a university in the United Arab Emirates. A total of 25 students with disabilities were recruited and selected using purposeful sampling methods. None of the participants refused to participate. The researchers recruited all study participants, and only students who experienced disability were included. The study participants were aged between 19 and 42 years old. Of 25 participants, 20 were males, and 5 were females. The most common disabilities among the student participants were learning difficulties, writing and reading difficulties, speech problems, hearing disabilities, and mobility impairments. Blindness and mobility issues were present in some of the participants, while other students struggled with their learning and writing. Most students who participated studied in the College of Humanities, Arts, and Social Sciences. For inclusion criteria, students were included if they had attended the university full-time or part-time and were at least 18 years old. In this study, we only recruited students with disabilities. A physician or psychiatrist must have determined that, as an individual with a disability, he/she has a physical or mental impairment. In this study, most of our participants experienced physical disabilities. They experienced learning, writing, reading, speech, hearing, blindness, and mobility problems. For exclusion criteria, students with severe anxiety or depression and those with severe mental health or psychiatric symptoms of their disabilities were excluded.

### Data collection

The primary data collection method was semi-structured interviews. All interviews were conducted at the Disability Resource Center at the University of Sharjah in UAE. The first, second, and third authors, PhD holders, and qualitative researchers collected the data. All interviewers are male faculty members at the University of Sharjah. Interviews were audiotaped using a digital recording device with participants’ consent. Each interview lasted between 35 and 55 minutes. In this study, data saturation was achieved at the 24th interview. Memos and field notes were also two components of the data collection techniques. These data collection methods were formed by triangulating data to improve the credibility of interpretations of the data ( Lincoln & Guba, 2017). These additional data collection methods were essential in guiding us in interpreting the participants’ experiences, feelings, perspectives, and beliefs. Before creating this interview guide, approval was received from the University of Sharjah’s ethical committee. Methodological methods should be adhered to while ensuring the quality assurance of an interview for a scientific application. We started our study by reading academic journals focusing on students with impairments, especially those attending universities. A face-validation procedure was conducted by a panel of three experts from various programs and disability centers at the University of Sharjah after the first draft of the interview guide was created. These specialists were experienced educators with a history of working with children with disabilities on research projects and in publications. Several changes were made to the interview guide in response to their suggestions. Before the interview, it is critical to ensure that the room in which the interview was held is fully accessible to wheelchair users and has sufficient space to allow the participants to move around while in the room.

### Data analysis

In this study, we adopt the content analysis approach ( Graneheim & Lundman, 2004) to analyze the data. Using this data analysis methodology, we could look into the differences and similarities within and across several data sets ( Graneheim & Lundman, 2004). In this study, NVivo software was used to manage the data. The audio recordings of each interview were attentively listened to and then transcribed immediately after the interview. To guarantee consensus and to ensure that everyone on the research team had an in-depth understanding of the topic, each interview’s transcript was read and reread several times. Then, themes resulting from the data were conceptualized, defined, and developed using open coding. All researchers agreed on the themes obtained after analyzing the codes derived from the interview data. Then, the relationships between the themes that had been extracted were determined using conceptual patterns and tables. We took the responses from the data related to the students’ experiences. All codes and units relevant to this study were analyzed and evaluated regarding their similarities and dissimilarities. Each study team member shaped the significant themes and subthemes, ultimately abstracted and categorized.

***Study rigor***

The study’s reliability was enhanced by gathering data and information from multiple sources, including interviews, field notes, and memoranda. We meticulously examined the data utilizing NVivo coding and examined each word and line in detail. Upon identifying areas of ambiguity during the data analysis process, we revisited a select group of student participants to validate and expand upon their viewpoints. Furthermore, five additional researchers (AS, RM, MS, FRA, and SAR) have independently verified the data analysis methods. The study published the criteria used to inform the researchers’ judgments and the process used to choose the study participants, thereby enhancing auditability. The research methodology and the results of our inquiry were meticulously recorded. We maintained a notebook to record the subjective choices and their corresponding reasons or rationales. The transferability of the study was guaranteed by presenting comprehensive details on the participants, setting, and study results, enabling readers to evaluate the study based on their own experiences.

## Results

In this study, we identified four distinct but connected themes that emerged from the interviews and data analysis that described the experiences of students with disabilities enrolling in a university: 1) perceptions about the university; 2) socialization and integration; 3) barriers to studying in the university; and 4) support in the university journey. In addition, theme 1 has one sub-theme. Theme 2 has two subthemes. Theme 3 has three subthemes, and theme 4 has eight subthemes.

### Theme 1: Perceptions about the university

***Subtheme: Positive experiences studying at the university***

Positive experience is an aspect that determines the success of students with disabilities in pursuing education at university. Generally, participants indicated that they had had positive experiences at the university.

*I have positive experiences from studying at UOS… In addition, the courses that talk about our general life and history provide us with many exciting and valuable courses. Also, … I meet my friends and colleagues daily, and I am very happy to have my friends. I am also satisfied with the help of the Disability Center and the interpreters. (Participant 3)*

***Subtheme: Negative experiences studying at the university***

However, some participants have had negative classroom experiences. According to one participant, he usually has problems with some professors.

*Among the problems I usually face during my studies, in general, are dealing with some professors … because some could not differentiate that not all students with disabilities are the same. At first, everything was simple, but now there are some challenges… (Participant 15)*

### Theme 2: Socialization and Integration

***Subtheme: Relationship with faculty members and staff***

Social interaction with others benefits the development of one’s abilities to communicate and interact. Study participants agreed that the university had a welcoming social atmosphere, which helped social integration. Participants discussed their interactions with peers and lecturers. They described having positive relationships with their professors.

*… Many professors know my condition; others around me also know it. As a result, things are simple, and the doctors usually deal with us and collaborate easily. Even though, sometimes, doctors give us an extra assignment… It is fine, and I am so happy at the university. (Participant 4)*

***Subtheme: Peer relationship***

Peer relationships are one factor determining student success in pursuing education. Activities on campus require harmonious relationships between students. We found that most participants had good relationships with their friends at the university. Some made connections and friends when they joined the university. Some students with disability communicate with friends with an interpreter.

*… Initially, joining the university was impossible, but I made new connections and friends when I joined. I could communicate with everyone, including professors and especially my friends, at any time, but I also communicated with the interpreter. Some students were close to me, so I have friends now… I have three best friends, and we share information… (Participant 17)*

### Theme 3: Barriers to the journey

Barriers to higher education for individuals with disabilities exist on many different levels. Each participant encountered numerous obstacles in university, including barriers to professors, exams, questions, and accessibility issues.

***Subtheme: Professors as a barrier***

Some participants indicated that professors are a barrier to their university journey. One participant said that he had a negative experience with his professor.

*During my undergraduate studies, I encountered a professor who did not understand my case, and to be honest, he disappointed me and caused my GPA to drop. Apart from hearing difficulty, I face many other complex problems. For example, some professors (two or three) needed help understanding my case…. (Participant 19)*

***Subtheme: Exams and levels of questions as barriers***

Study participants indicated that exams and levels of questions were barriers. They struggled with exams at the university, especially when they written questions. One study participant indicated that exams (quizzes, midterms, and final exams) are barriers during their education at the university.

*… Sometimes, I struggle with exams. However, I should not take these as a barrier that may stop me from completing my journey. In general, I see that our exams are so challenging. During the exams sometimes, it is tough to solve the exams. My challenge was when I had to solve written questions… (Participant 18)*

***Subtheme: Campus accessibility as a barrier***

Campus accessibility is essential for students with disabilities. Some students indicated that they have problems with parking spaces. According to a participant:

*I find parking in certain places makes me struggle with… parking is complicated. Wheelchair. Especially during exams, some non-students with disabilities park in our parking. I need space because I am a student with disability and mobility impairment and I need space … I hope that we have special parking for deaf students. **(**Participant 2)*

### Theme 4: Supports to the journey

Even though students with disabilities encounter many challenges or other difficulties along the way, the majority of participants said they receive support. Their journeys’ success correlates with university facilities, academic performance, and parental support. Interviews with students demonstrated that institutional assistance encourages academic persistence among students with disabilities

***Subtheme: University Facilities support***

In our interview, we found that accessibility to university facilities is essential for students with disabilities. One of the participants indicated that all the facilities in the university are accessible or easy for them to use.

*…All the facilities in the university are accessible, so I have no issue with reaching the university’s facilities. I do not run into any issues. No problem with facilities… everything goes as I plan. I did not face any difficulties while attending the campus. I was grateful for every time I entered the facilities. (Participant 12)*

***Subtheme - Disabilities Center service’s support***

The university’s support services for students with disabilities were essential in guaranteeing that students could succeed academically. The student has benefited from the Disability Resources Center, which helped, advised, and supported the university’s students. Students indicated they needed to ensure they had access to these disability services.

*Disability Resources Center has helped me a lot. They advised, guided, and facilitated everything for me and my classmates. Also, they encouraged me… and told me what I should gain from the university and what goals I need to achieve throughout the university years. (Participant 17)*

***Subtheme: Academic and Curriculum support***

Participants acknowledged that their professors were essential to their academic performance. Students repeatedly stated that professors’ responses and knowledge can affect access and equity. Participants realized that they were not rejected or refused when they requested assistance. An autistic participant described his encounters with his lecturers as mostly favorable experiences.

*I am glad to see my professor in the classroom. I am grateful for his constant support. Like one of my professors, he supports me a lot. We succeeded. Like, none of my instructors ever actually objected to me… (Participant 10)*

***Subtheme: Professor’s support***

Educators or professors in the classroom constantly assisted the students. According to participants, university professors give full attention and support to the students and are willing to help them, even when they are sick, unable to attend, or absent.

*The professors were good, and because they supported me, everything became more accessible. Dr. AA helped us a lot. Also, Dr. YN and Dr. AS (faculty members) … help me and solve my problem immediately when I see them. They assisted and supported us well. I am so happy, and I was trying as much as possible to do both theory and practice and focus on studying and attending my classes. (Participant 14)*

***Subtheme: Counseling support***

Participants indicated that a counseling center is essential for students with a disability at the university. Participants reported that the university counseling center offers stress management services to support stress-reduction methods. They receive problem-solving instruction from the school’s psychologists on how to handle difficulties. Participating in campus stress management programs helped students prepare for stressful classes and other campus activities.

*Yes, counseling center …. It helps me with stress management. They (counselors) provide relaxation exercises and other stress management techniques. There are some problems, and I attend stress management programs to overcome my challenges. It works very well. (Participant 12)*

***Subtheme: Peer support***

Peer support is essential for students with disabilities. Students indicated that they have support from their friends. Working together in groups, for example, requires support from other students, especially non-students with disabilities.

*I usually ask my colleagues, the hearing-impaired students, and other friends to assist me with my academics when I do not comprehend something. Even people who are not disabled would try to help me. We have a WhatsApp group where we assist one another by notifying, updating, and contacting each other when something new occurs… Members of this group regularly converse, translate, and interpret everything being sent. (Participant 4)*

***Subtheme: Family support***

Students with disabilities face challenges during their university journey, and support from their family members will be necessary. Study participants indicated that they have support from their family members. One of the participants indicated that their family supports them.

*… I want to say thanks to my wife. My wife is also deaf, so things were easy, and she helped me with my studies. I used to think it was impossible to study at university, but my wife constantly encouraged and supported me. Although things started hard, they improved over time, and I am grateful to those who supported me… (Participant 13)*

***Subtheme: Financial Support***

Because they are eligible for scholarships, students are satisfied to attend the university. Financial aid helps these students pay for some of their tuition, handouts, and textbooks. Several participants said they stay in school when their financial needs are satisfied. The college supports some students with disabilities by providing financial aid. Some students mentioned getting financial assistance to cover some of their tuition costs.

*The financial aid I receive enables me to purchase textbooks, handouts, and a portion of my tuition without worrying about where I will find the money to do so… Yes, I am glad to study at the university and get the scholarship… I would like to take this opportunity to thank H.H SJ [Queen] and sheik Dr. SQ [king) of S Emirate] for this opportunity and scholarship. (Participant 11)*

## Discussion

Student with disabilities constitute a significant and growing minority group in higher education, contributing to the overall variety of the student population ( Römhild & Hollederer, 2023; Mottershead et al., 2015). Our study results indicated that students have positive perceptions of studying at the university and indicated an excellent perception of the university. These findings support Clavijo Castillo and Bautista-Cerro (2020), who found that higher education is essential to building a learning environment that can contribute equality solutions to difficulties for those with disabilities in the educational setting. We also found in our study that some students had a negative perspective and impression of the university. The perceptions of students with disabilities on the services and initiatives offered by universities are crucial factors that influence these students’ performance ( Cawthon & Cole, 2010). Most students who had issues with university teachers nonetheless expressed satisfaction with their college experience ( Yssel et al., 2016). A student’s sense of belonging and capacity for self-advocacy contribute to better satisfaction and retention ( Fleming et al., 2017).

Study participants agreed that the university had a welcoming social atmosphere, which helped social integration. Similar to our findings, integrating classmates and educators, campus activities, and extra-curricular activities for students with disabilities increased success in school ( Accardo et al., 2019). The participation of students with disabilities in social activities promotes successful academic integration. According to Choi et al. (2019), university students with disabilities who are academically and socially integrated are more likely to stay in school and less likely to drop out. In this context, academic integration entails communication with and assistance from staff members and educators ( Römhild & Hollederer, 2023). In this study, participants shared their positive experiences regarding relationships with faculty members. Other studies noted similar findings to our results. For example, disability-aware professors frequently assist students with disabilities in learning, solving problems, and passing classes with little to no difficulties ( Mutanga & Walker, 2017; Timmerman & Mulvihill, 2015). When educators know how to help students, they can experience favorable social implications ( Agarwal et al., 2015). The best opportunity for successful academic outcomes for students with disabilities is to form positive relationships with their teachers and peers ( Mutanga & Walker, 2017).

The participants’ relationships included interactions with peers, faculty, and staff. Study results indicated that participants have good relationships with their friends. When participants had positive contact with their colleagues, they felt a sense of belonging. These interactions also help students with disabilities feel successful. Some studies show difficulties when disabled university students interact socially with peers ( Nel et al., 2015; Schwab et al., 2018). Stereotypical threats, stigmatizations, and marginalization are among the difficulties students face with social participation ( Desombre et al., 2018; Subu et al., 2023). According to French (2017), university students with disabilities have a sense of belonging when they connect favorably with fellow students and teachers during and after class. When their peers know how to help them, students with disabilities can experience favorable effects in society ( Agarwal et al., 2015).

Our study results indicated that students with disabilities experienced several challenges during their journey at the university. Higher education is crucial for building an academic environment that can contribute equality solutions to challenges for students with disabilities in inclusive learning environments ( Clavijo Castillo & Bautista-Cerro, 2020). A study indicated that it is highly challenging for individuals with disabilities to enroll in a university ( Fernández-Batanero et al., 2022). In contrast to non-students with disabilities, other studies have indicated that academic distress, a lack of social support, and a lack of campus involvement are challenges ( Fleming et al., 2017). Disabled university students who attend universities employ coping mechanisms like self-awareness, self-advocacy, and tenacity and resilience to deal with academic difficulties ( Hengen & Weaver, 2018; Ju et al., 2017).

Other studies highlight other learning challenges. The absence of training for instructors to employ an approach that encourages inclusion in the classroom by their students’ requirements stands out among them ( Heiman et al., 2017). Our findings align with another study on how these students do not receive teacher preparation in higher education ( Nimante et al., 2021). Additionally, students are informed how difficult it is to get material resources because, for the most part, they are either inadequate or unsuitable for their needs ( Alsalem & Abu Doush, 2018; Dreyer, 2021). Our study results also indicated that exams (quizzes, midterm, and final exams) are barriers during their education at the university. Our participants found that questions during exams are difficult for them regarding level. The results of this study are generally in line with those of another study that found that students with disabilities encountered significant educational challenges related to tests and information access because of a lack of support ( Kisanga, 2020).

The university’s services for student with disabilities ensured their success there. Campus accessibility is essential for students with a disability at the university. Study results indicated that some students have a problem related to parking space, and other participants have difficulty accessing sidewalks. This result is in line with a study in which students with disabilities complained about the inadequate infrastructure and facilities at the university, as well as their difficulty accessing educational resources as a result of these problems ( Nel et al., 2015). As these challenges can impede accessibility for these students, students with disabilities exhibit educational demands that must be addressed to attend education successfully ( Braun & Naami, 2019). The results of this study are also in line with another study’s findings, which found that students with disabilities encountered considerable educational challenges related to accessibility ( Kisanga, 2020).

Additionally, conventional architectural difficulties such as classrooms, stairs, insufficient auditoriums, heavy doors, malfunctioning elevators, or the lack of ramps and signage continue to be barriers to higher education ( García-González et al., 2020). Accessibility to the university facilities is essential for students with disabilities. Study results indicated that participants indicated that all the facilities in the university are accessible or easy for them to use. Participants talked about how the school’s learning support programs encourage them to persevere, challenge them to explore options, and help them stay on track academically. Similar to this finding, university settings support the persistence of students with disabilities by enhancing their learning and academic performance, offering persistence programs like stress management and student success training, providing them with aid, and equipping them with resources ( Mamiseishvili & Koch, 2012).

The study’s findings showed that the university’s disabilities center offers support to students, and it is crucial for them to make sure they have access to these services before beginning their academic studies. Previous studies have demonstrated that disability-related services, such as disability offices, positively impact graduation intentions and grade point averages ( Singley, 2017). According to Brophy-Felbab (2021), disability-related services are crucial for student retention and performance. However, no appreciable associations exist between grade point average and disability-related services ( Santos et al., 2019). Study participants claimed that the university program accommodated and enhanced their learning despite their particular limitations. According to the study’s findings, participants agreed that their educators were crucial to their academic success. It can be challenging for many students with disabilities to benefit from essential initiatives that improve learning outcomes ( Boney et al., 2019). Similar issues with curriculum design are among the difficulties colleges face as they deal with the disability issue ( Everett & Oswald, 2018). Developing inclusive learning materials should receive more vital support from teaching staff and curriculum designers ( Fleet & Kondrashov, 2019), with universal design serving as a common framework for such efforts ( Grier-Reed & Williams-Wengerd, 2018).

Study results indicated that professors at the university provide full attention and support to the students, and they are all willing to support them. Similar to these results, educators provide support to students with disabilities. The principle of inclusion depends on academics who value and assist students with disabilities in the classroom. Participants in this study reported that faculty members kept their attention during and after class by offering them opportunities to explore, ask questions, and work out problems. Most of the class is ensured to participate through discussion, demonstrations, and questions. According to Francis et al. (2019), instructors’ negative attitudes about disability are the cause of the mistrust and cynicism that exist between students with disabilities and their educators. Faculty members aware of disability concerns are better equipped to give students with disabilities the help they need during the teaching and learning process ( Harnett, 2016). To succeed, students with disabilities need peer support. For instance, working in a group requires the assistance of other students, especially non-students with disabilities. Our research shows that during class discussions and project presentations, peers assist students in finding answers to questions. Students with disabilities retain positive relationships with their classmates and exchange unique and crucial information during and after lecture hours, which is consistent with our findings ( O’Shea & Meyer, 2016).

In this study, participants stated they would remain in the program until their financial necessities were met. The university provides financial support for specific numbers of its students with disabilities. A few students claimed they were receiving financial aid to help with the expense of their tuition. Some participants indicated that receiving financial aid allowed them to get intellectually assimilated into the institution since it allowed them to purchase study materials, learn, and study in groups. We found that our participants indicated that their family supports them. They benefited from their relationships during university, which made for a better higher education experience. It has been recognized that providing these individuals with equal opportunity to non-disabled people in families and the greater community has a positive effect on the education of children with disabilities ( Ceka & Murati, 2016). Although family and partner support does not directly correlate with academic success, it does increase students’ overall satisfaction ( Lombardi et al., 2016). Parents who supported the idea of higher education for all—both literate and uneducated—supported and promoted their disabled children’s university education ( Hegna & Smette, 2017).

### Study limitation

The participants in this study only represented one university in the United Arab Emirates, which was the main limitation. First, inclusive education approaches for students with disabilities may vary among universities in the UAE. The limitation of qualitative research, like this study, is that it cannot be generalized. The generalizability of this study’s findings outside this particular institution can be problematic due to its exclusive focus on the experiences of students with disabilities within this institution. Despite these limitations, this research has shed light on how inclusive policies and practices are perceived and understood in higher education institutions in the UAE. A larger population sample should be used to have more significance and beneficial implications. In addition, using a range of geographic locations would be advantageous for this research issue.

## Conclusion

This study shares the stories of students with disabilities and their experiences at the university. To accommodate these students, universities must modify their educational programs and offer services consistent with best practices. To enable these students to attend classes at university, a variety of infrastructure, teaching-learning, and institutional management-related factors are required. Promoting the success of students with disabilities in university education will only be possible by making higher education institutions more accessible, providing training for university faculty, and increasing community awareness of inclusive education in higher education. Enhancing the function of student support centers, which have the trained personnel and tools required to support the education of students with disabilities, will result in better implementation of inclusive education methods. This study looked into a specific instance of inclusive education techniques being applied to students with disabilities in a model higher education environment in the UAE rather than making a broad conclusion. Additionally, the study recommends modifying the physical, social, and educational environments to support the education of students with disabilities. Our research also indicates that to promote equity, university faculty members and staff need to be trained to assist students with disabilities. Implementing instructional strategies grounded in design for Learning may remove future challenges to learning, benefiting not just students with functional diversity but also other students. Enhancing instruction for students with disabilities has a favorable effect on education for all students. This study highlights some topics that need more significant investigation to develop best practices and a more profound knowledge of students with disabilities and to create a more inclusive environment for them. Further studies may provide additional context for these results, which may be applied to the entire student population with disabilities. Research on disability identification, faculty perspective, and the efficacy of involvement is especially recommended. With the results of this further research, attempts to provide a more equal education for all children will significantly increase the inclusion of students with disabilities. Future research should use a more diverse sample, involve academics and students, and emphasize the students’ experiences to get data from various perspectives and experiences.

## Ethical consideration and consent

We realized that for this study, ethics approval is necessary. Research Ethics Committee (REC) of the University of Sharjah approved this study (Reference number: REC-21-09-22 (VERSION 2) on 10 ^th^ September 2022. All study participants were made aware of the research’s purpose and methodology. We followed specific methods to obtain consent from visually impaired participants. Before data collection, the appropriate consent form was presented orally to the participants. We also used an audio recording of the informed consent document. The guidance for obtaining and documenting informed consent for deaf participants was also followed for those who cannot read or write. Hearing-impaired participants may need an interpreter present. To ensure privacy and comfort, we selected interview environments that could provide a quiet, private space to make all participants feel comfortable and confident. We made sure that students with disability who participated in this study felt secure during the interview. To preserve confidentiality, we provided information about the aim and method of the research to all participants with written informed consent, and they were assured of anonymity and confidentiality. Participants in the study must be assured that their data is kept anonymous unless they give their full consent.

## Short authors’ biography

**Muhammad Arsyad Subu:** Specialization: Mental health nursing. Research: Mental health nursing and nursing education. **Richard Mottershead:** Specialization: Mental health nursing. Research: Mental health interventions and social care needs. **Ahmed Mahmoud Sallam**: Specialization: Counseling psychology. Research: Students with disabilities, rehabilitation therapies. **Jacqueline Maria Dias**: Specialization: Oncology and geriatrics nursing. Research: Curriculum design and evaluation simulation & clinical teaching and learning. **Janisha Kavumpurath.** Specialization: Adult nursing. Research: Basic life support among adults. **Fatma Refaat Ahmed.** Specialization: Critical care and emergency nursing. Research: critical care nursing and critical care hematology nursing. **Heba Hesham Hijazi.** Specialization: Public health management and policy. Research: Quality of healthcare services, health policies, and utilization of healthcare services. **Mini Sara Abraham**: Specialization: Medical Surgical Nursing. Research: Nursing administration quality COPD. **Alounoud Mohamed Al Marzouqi**. Specialization: Health care management, quality, excellence. Research: Leadership & health information management. **Vidya Seshan:** Specialization: Maternal Health. Research: Women’s and Maternal Health. **Shahd Mohammed Elabed:** Health care management. Research: Management of infectious diseases. **Ahmad Rajeh Saifan.** Specialization: Emergency nursing education. Research: Diabetes in adults, emergency hospitalization. **Syed Azizur Rahman**. Specialization: Health care delivery system, public health, and health care evaluation. **Nabeel Al-Yateem.** Specialization: Pediatric nursing. Research: Children with chronic illness and school nursing care.

## Data Availability

Derived data supporting this study’s findings are available from the corresponding author [RM] upon reasonable request. The data is restricted due to institutional confidentiality policies pertaining to student information.

## References

[ref1] Accardo AL Kuder SJ Woodruff J : Accommodations and support services preferred by college students with autism spectrum disorder. *Autism: The International Journal of Research and Practice.* 2019;23(3):574–583. 10.1177/1362361318760490 29475372

[ref2] Agarwal N Moya EM Yasui NY : Participatory action research with college students with disabilities: Photovoice for an inclusive campus. *Journal of Postsecondary Education and Disability.* 2015;28:243–250. Reference Source

[ref3] Algolayalt AS Alodat AM Muhaidat MA : Perspectives of Students with Disabilities on Inclusive Education Challenges in Higher Education: A Case Study of a Jordanian University. *TEM Journal.* 2023;12(1):406–413. 10.18421/TEM121-50

[ref4] Alsalem GM Abu Doush I : Access Education: What is needed to have accessible higher education for students with disabilities in Jordan? *International Journal of Special Education.* 2018;33:541–561. Reference Source

[ref5] Aquino KC : A New Theoretical Approach to Postsecondary Student Disability: Disability-Diversity (Dis)Connect Model. *Journal of Postsecondary Education and Disability.* 2016;29(4):317–330. Reference Source

[ref6] Boney JD Potvin MC Chabot M : The goals program: Expanded supports for students with disabilities in postsecondary education (practice brief). *Journal of Postsecondary Education and Disability.* 2019;32(3):321–329. Reference Source

[ref7] Braun AMB Naami A : Access to higher education in Ghana: Examining experiences through the lens of students with mobility disabilities. *International Journal of Disability, Development and Education.* 2019;1-21. 10.1080/1034912X.2019.1651833

[ref8] Brewer G Urwin E Witham B : Student with disability experiences of Higher Education. *Disability & Society.* 2023;1–20. 10.1080/09687599.2023.2263633

[ref9] Brophy-Felbab S : *Community college success of students with disabilities* (Order No. 28321182). Available from ProQuest One Academic; Publicly Available Content Database. (2506585956). 2021. Reference Source

[ref10] Burgstahler SE : *Creating inclusive learning opportunities in higher education: A universal design toolkit.* Harvard Education Press;2020.

[ref11] Carroll JM Pattison E Muller C : Barriers to bachelor’s degree completion among college students with a disability. *Sociological Perspectives.* 2020;63(5):809–832. 10.1177/0731121420908896 33795909 PMC8009488

[ref12] Cawthon SW Cole EV : Postsecondary Students Who Have a Learning Disability: Student Perspectives on Accommodations Access and Obstacles. *Journal of Postsecondary Education and Disability.* 2010;23(2):112–128. Reference Source

[ref13] Ceka A Murati R : The Role of Parents in the Education of Children. *Journal of Education and Practice.* 2016;7(5):61–64. Reference Source

[ref14] Choi AN Curran GM Morris EJ : Pharmacy students’ lived experiences of academic difficulty and Tinto’s theory of student departure. *American Journal of Pharmaceutical Education.* 2019;83(10):2150–2160. 10.5688/ajpe7447 32001879 PMC6983888

[ref15] Clavijo Castillo RG Bautista-Cerro MJ : Inclusive education. Analysis and reflections in Ecuadorian Higher Education. ALTERIDAD. *Educational Review.* 2020;15(1):113–124. 10.17163/alt.v15n1.2020.09

[ref16] Creswell JW : *Qualitative inquiry & research design.* 3rd ed. Sage Publications, Inc.;2012.

[ref17] Creswell JW Poth CN : *Qualitative inquiry & research design: Choosing among five approaches.* 4th ed. Sage Publications, Inc.;2018.

[ref18] Desombre C Anegmar S Delelis G : Stereotype threat among students with disabilities: The importance of the evaluation context on their cognitive performance. *European Journal of Psychology of Education.* 2018;33(2):201–214. 10.1007/s10212-016-016-0327-4

[ref19] Dreyer LM : Specific learning disabilities: Challenges for meaningful access and participation at higher education institutions. *Journal of Education.* 2021;85:1–18. 10.17159/2520-9868/i85a04

[ref20] Ehlinger E Ropers R : "It’s all about learning as a community": Facilitating the learning of students with disabilities in higher education classrooms. *Journal of College Student Development.* 2020;61(3):333–349. 10.1353/csd.2020.0031

[ref21] Erickson W Lee C Schrader S von : *2013 Disability Status Report: United States.* Cornell University Employment and Disability Institute (EDI);2014. Reference Source

[ref22] Everett S Oswald G : Engaging and training students in the development of inclusive learning materials for their peers. *Teaching in Higher Education.* 2018;23(7):802–817. 10.1080/13562517.2017.1421631

[ref23] Fernández-Batanero JM Montenegro-Rueda M Fernández-Cerero J : Access and Participation of Students with Disabilities: The Challenge for Higher Education. *International Journal of Environmental Research and Public Health.* 2022;19(19):11918. 10.3390/ijerph191911918 36231217 PMC9565787

[ref24] Fleming AR Plotner A Oertle KM : College Students with Disabilities: The Relationship between Student Characteristics, the Academic Environment, and Performance. *Journal of Postsecondary Education and Disability.* 2017;30(3):209–221. Reference Source

[ref25] Fleet C Kondrashov O : Universal design on university campuses: A literature review. *Exceptionality Education International.* 2019;29(1):136–148. Reference Source

[ref26] Francis GL Duke MJ Fujita M : It’s a constant fight: Experiences of college students with disabilities. *Journal of Postsecondary Education and Disability.* 2019;32(3):247–261. Reference Source

[ref27] French A : Toward a new conceptual model: Integrating the social change model of leadership development and Tinto’s model of student persistence. *Journal of Leadership Education.* 2017;16(3):97–117. 10.12806/V16/I3/T1

[ref28] Gaad E Almotairi M : Inclusion Of Student with Special Needs Within Higher Education In UAE: Issues and Challenges. *Journal of International Education Research (JIER).* 2013;9(4):287–292. 10.19030/jier.v9i4.8080

[ref29] García-González JM Gutiérrez Gómez-Calcerrada S Solera Hernández E : Barriers in higher education: Perceptions and discourse analysis of students with disabilities in Spain. *Disability & Society.* 2020;36(4):579–595. 10.1080/09687599.2020.1749565

[ref30] Graneheim UH Lundman B : Qualitative content analysis in nursing research: concepts, procedures, and measures to achieve trustworthiness. *Nurse Educ. Today.* 2004;24(2):105–112. 10.1016/j.nedt.2003.10.001 14769454

[ref31] Grier-Reed T Williams-Wengerd A : Integrating universal design, culturally sustaining practices, and constructivism to advance inclusive pedagogy in the undergraduate classroom. *Education Sciences.* 2018;8(4):167–181. 10.3390/educsci8040167

[ref32] Harnett M : *An exploration of the personal journeys of student with disabilitys during the first year of Higher Education.* Cardiff, UK: Cardiff Metropolitan University;2016. (Doctoral dissertation). Reference Source

[ref33] Hegna K Smette I : Parental influence in educational decisions: young people’s perspectives. *British Journal of Sociology of Education.* 2017;38(8):1111–1124. 10.1080/01425692.2016.1245130

[ref34] Heiman T Fichten CS Olenik-Shemesh D : Access and perceived ICT usability among students with disabilities attending higher education institutions. *Education and Information Technologies.* 2017;22:2727–2740. 10.1007/s10639-017-9623-0

[ref35] Hengen S Weaver AD : Post-secondary students with disabilities: Increasing self-advocacy through educational plan participation. *The School Psychologist.* 2018;72(2):7–18.

[ref36] Ju S Zeng W Landmark LJ : Self-Determination and Academic Success of Students with Disabilities in Postsecondary Education: A Review. *Journal of Disability Policy Studies.* 2017;28(3):180–189. 10.1177/1044207317739402

[ref37] Kisanga SE : Coping with educational barriers in Tanzanian inclusive education settings: Evidence from students with sensory impairment. *Current Psychology.* 2020;41(7):4750–4759. 10.1007/s12144-020-00977-w

[ref38] Lincoln Y Guba EG : *The SAGE Handbook of Qualitative Research.* Newbury Park, CA: SAGE Publications Inc;2017.

[ref39] Lombardi A Murray C Kowitt J : Social support and academic success for college students with disabilities: Do relationship types matter? *Journal of Vocational Rehabilitation.* 2016;44:1–13. 10.3233/JVR-150776

[ref40] Magnus E Tøssebro J : Negotiating individual accommodation in higher education. *Scandinavian Journal of Disability Research.* 2014;16(4):316–332. 10.1080/15017419.2012.761156

[ref41] Mamiseishvili K Koch LC : Students with disabilities at 2-year institutions in the United States: Factors related to success. *Community College Review.* 2012;40(4):320–339. 10.1177/0091552112456281

[ref60] Mottershead R Kumardhas V Masulani Mwale C : Mental health within different ethnic groups and minorities. *Mental Health Across the Lifespan.* Steen M Thomas M , editors. Routledge;2015; pp.46–59. 10.4324/9781315777573

[ref42] Mutanga O Walker M : Exploration of the academic lives of students with disabilities at South African universities: Lecturers’ perspectives. *African Journal of Disability.* 2017;6:1–9. 10.4102/ajod.v6i0.316 28730069 PMC5502474

[ref43] National Center for Education Statistics: *Students with disabilities.* U.S. Department of Education, Institute of Education Sciences;2022. Reference Source

[ref44] Nel K Rankoana SA Govender I : The challenges experienced by students with a physical disability (SWPD) at a higher education institution in South Africa. *African Journal for Physical, Health Education Recreation and Dance.* 2015;1(4):801–811. Reference Source

[ref45] Nimante D Baranova S Stramkale L : The university administrative staff perception of inclusion in higher education. *Acta Paedagogica Vilnensia.* 2021;46:90–104. 10.15388/ActPaed.2021.46.6

[ref46] O’Shea A Meyer R : A Qualitative Investigation of the Motivation of College Students with Nonvisible Disabilities to Utilize Disability Services. *The Journal of Postsecondary Education and Disability.* 2016;29:5–23.

[ref47] Ostiguy-Finneran B Peters ML : Ableism. *Readings for Diversity and Social Justice.* Adams , editors. New York: Routledge;2018.

[ref48] Prohn SM Kelley KR Westling DL : Supports’ perspectives on the social experiences of college students with intellectual disability. *Inclusion.* 2019;7(2):111–124. 10.1352/2326-6988-7.2.111

[ref49] Römhild A Hollederer A : Effects of disability-related services, accommodations, and integration on academic success of students with disabilities in higher education. A scoping review. *European Journal of Special Needs Education.* 2023;1–24. 10.1080/08856257.2023.219507

[ref50] Santos SBD Kupczynski L Mandy M : Determining Academic Success in Students with Disabilities in Higher Education. *International Journal of Higher Education.* 2019;8(2):16–38. 10.5430/ijhe.v8n2p16

[ref51] Schwab S Nel M Hellmich K : Social participation of students with special educational needs. *European Journal of Special Needs Education.* 2018;33(2):163–165. 10.1080/08856257.2018.1424784

[ref52] Singley DM : *Effects of academic coaching on college students with learning disabilities or attention-deficit hyperactivity disorder (Order No. 10278516).* ProQuest One Academic;2017. (1914924962). Reference Source

[ref53] Subu MA Wati DF Al-Yateem N : ‘Family stigma’ among family members of people with mental illness in Indonesia: a grounded theory approach. *International Journal of Mental Health.* 2023;52(2):102–123. 10.1080/00207411.2021.1891363

[ref61] Subu MA Dias JM Mottershead R : Exploring mental health stigma among Indonesian healthcare students towards individuals with mental illnesses: a qualitative study. *International Journal of Qualitative Studies on Health and Well-being.* 2024;19(1): 2327103. 10.1080/17482631.2024.2327103 PMC10930137 38465669

[ref54] Taneja-Johansson S : Facilitators and barriers along pathways to higher education in Sweden: A disability lens. *International Journal of Inclusive Education.* 2021;1–15. 10.1080/13603116.2021.1941320

[ref55] Timmerman LC Mulvihill TM : Accommodations in the College Setting: The Perspectives of Students Living with Disabilities. *Qualitative Report.* 2015;20(10):1609–1625. 10.46743/2160-3715/2015.2334 Reference Source

[ref56] UNESCO: *Education and disability: Analysis of data from 49 countries.* Author;2018. Reference Source

[ref57] United Arab Emirates Government: Education for people of determination. 2023. Reference Source

[ref58] Yssel N Pak N Beilke J : A door must be opened: Perceptions of students with disabilities in higher education. *International Journal of Disability, Development and Education.* 2016;63:384–394. 10.1080/1034912X.2015.1123232

[ref59] Zhang Y Rosen S Cheng L : Inclusive Higher Education for Students with Disabilities in China: What Do the University Teachers Think? *Higher Education Studies.* 2018;8(4):104–115. 10.5539/hes.v8n4p104

