# Supplementary material for: Experiences of Students with Disabilities Enrolling in Higher Education: A Qualitative Study in the United Arab Emirates
Source: F1000Res. 2025 Jan 2;14:14. [Version 1] doi: 10.12688/f1000research.158481.1 (PMC11736105; doi:10.12688/f1000research.158481.1)
Supplement: Peer review history

# Reviewer report: Reviewer response for version 1

**DOI:** 10.5256/f1000research.174076.r356051

**Published:** 2025-01-15

**Authors:** Dr. Sarah Sanad

**Affiliations:** 1 College of Nursing, Dubai Medical University, Dubai, Dubai, United Arab Emirates

The manuscript titled "Experiences of Students with Disabilities Enrolling in Higher Education: A Qualitative Study in the United Arab Emirates" explores the lived experiences of students with disabilities in a higher education setting. Using a qualitative descriptive research approach, the study identifies barriers and facilitators influencing the academic and social integration of these students. The research draws on semi-structured interviews with 25 participants, revealing themes such as perceptions of the university, socialization, barriers, and support systems. The findings highlight the need for more inclusive practices and institutional modifications to support students with disabilities.

 The manuscript is well structured and provides a coherent narrative of the study. The cited literature is well presented and current. The study design is appropriate and sound for a descriptive qualitative study that includes a small number of participants. It is recommended that more information is provided on the interview guide, its development and validation. The data is available upon request from PI, which is acceptable considering the ethical guidelines for students’ data management. Anonymized excerpts or summaries of interview data can be provided as supplementary material while maintaining complete student confidentiality.

 The conclusions drawn are adequately supported by the results. The themes extracted are supported by the data and are tied appropriately to the implications of the findings.

 Recommendations:

 To elaborate on the interview methodology

Is the work clearly and accurately presented and does it cite the current literature?

Yes

If applicable, is the statistical analysis and its interpretation appropriate?

Not applicable

Are all the source data underlying the results available to ensure full reproducibility?

Partly

Is the study design appropriate and is the work technically sound?

Yes

Are the conclusions drawn adequately supported by the results?

Yes

Are sufficient details of methods and analysis provided to allow replication by others?

Yes

Reviewer Expertise:

Nursing profession and image, Public health, Simulation-based teaching and learning, Leadership and management in healthcare, Critical care and emergency nursing, Disaster management, E-learning and hybrid learning, Pedagogies in teaching and learning, Students’ perceptions and experiences in education, Quality of work life, Job satisfaction among nurses, Risk perception, Mental health during crises, Psychometric evaluation of healthcare tools, Impact studies, Quality of life, Health promotion, Awareness campaigns.

I confirm that I have read this submission and believe that I have an appropriate level of expertise to confirm that it is of an acceptable scientific standard.

# Response

**Published:** 2025-01-16

**Authors:** Dr. Richard Mottershead

**Affiliations:** College of Health Sciences, University of Sharjah, Sharjah, Sharjah, United Arab Emirates

The research team would like to collectively thank the reviewer for their review and the time taken to undertake this task which adds credibility and rigor to the article.

# Reviewer report: Reviewer response for version 1

**DOI:** 10.5256/f1000research.174076.r356054

**Published:** 2025-01-13

**Authors:** Dr. Glen Ford Valdez

**Affiliations:** 1 Shaqra University, Riyadh Province, Saudi Arabia

Thank you for the opportunity to review this fascinating research project. The manuscript was well written and well structured, and the methodology is systematic and accurate, with only some questions that require clarification. 

 1. I understand that the researchers are experts in their field, but was there special training conducted among proponents to ensure accurate and cohesive delivery and execution of the interview sessions? 

 2. UAE was the setting of the study. I believe various nationalities reside or study at SU; was English used as the interview medium? How did the researchers address language levels or comprehension? If translations were done into Arabic languages, how would they be processed? 

 These are the only two questions I have. The rest were addressed in the study limitations.

Is the work clearly and accurately presented and does it cite the current literature?

Yes

If applicable, is the statistical analysis and its interpretation appropriate?

Not applicable

Are all the source data underlying the results available to ensure full reproducibility?

Yes

Is the study design appropriate and is the work technically sound?

Yes

Are the conclusions drawn adequately supported by the results?

Yes

Are sufficient details of methods and analysis provided to allow replication by others?

Yes

Reviewer Expertise:

Nursing Education, Nursing Leadership and Management, Population Health, Obesity , Artificial Intelligence in Healthcare

I confirm that I have read this submission and believe that I have an appropriate level of expertise to confirm that it is of an acceptable scientific standard.

# Response

**Published:** 2025-01-14

**Authors:** Dr. Richard Mottershead

**Affiliations:** College of Health Sciences, University of Sharjah, Sharjah, Sharjah, United Arab Emirates

Collectively the research team would like to thank the reviewer for their valuable time and input into our article. 

 To answer their questions the team is an international conglomeration of researchers from diverse backgrounds and this allowed us to utilize a diverse skillset to engage with the participants who were equally diverse. Collectively, we met and reviewed the a process for data collection that could be replicated and could be standardized. 

 Thank you for your time 

 Dr. Richard Mottershead
